# The complete chloroplast genome sequence of China *Lindera praecox* (Lauraceae) and intra-species diversity

**DOI:** 10.1080/23802359.2021.1944829

**Published:** 2021-07-01

**Authors:** Jiaojun Yu, Zhiliang Li, Hongjin Dong

**Affiliations:** a Hubei Key Laboratory of Economic Forest Germplasm Improvement and Resources Comprehensive Utilization, Huanggang Normal University, Huanggang, China; b Hubei Collaborative Innovation Center for the Characteristic Resources Exploitation of Dabie Mountains, Huanggang, China

**Keywords:** *Lindera*, complete chloroplast genome Lauraceae phylogeny

## Abstract

*Lindera praecox* is a signature composition in the broadleaved deciduous forest of East China and Japan. Presently, the complete chloroplast (cp) genome of this species was sequenced, assembled, and annotated. It is 152,818 bp in length and encodes 85 protein-coding genes, 36 transfer RNA (tRNA) genes and eight ribosomal RNA (rRNA) genes. The phylogenetic analysis indicated intraspecific varieties within *L. praecox* species collected in China and Japan. This chloroplast genome sequencing offers genetic background for resources conservation and phylogenetic studies.

*Lindera praecox* (Siebold & Zuccarini) Blume is the member with extremely size of fruit in the genus *Lindera*, distributed in the broadleaved deciduous forest of Hubei, Anhui, Zhejiang and Japan, as a diagnostic component (Li et al. 2008). In present study, the completed chloroplast genome sequence of *L. praecox* is reported contributing for better understanding its evolution and population genetics, and providing significant information for the phylogeny of Lauraceae.

Genomic DNA was extracted from fresh leaves of a seedling of *L. praecox* from Bodaofeng, Luotian, Hubei, China (115.5865992°, 31.1391175°, 928 m, in the valley; *Dong Hongjin* et al. *1272*, 2020-7-28; HIB, HTGC), the total genomic DNA was isolated according to a modified CTAB method (Doyle and Doyle 1987). Total genome DNA of *L. praecox* was sequenced by Illumina Hiseq 2500 Sequencing System (Illumina, Hayward, CA) to construct the shotgun library and assembled through the GetOrganelle software (Jin et al. 2020). The complete chloroplast genome of *L. praecox* was annotated with software PGA (Qu et al. 2019) and Geneious ver. 10.1 (Matthew et al. 2012) (http://www.geneious.com), and then submitted to GenBank (accession no. MW774641). The genome annotation was performed by aligning with the cp genomes of relatively related species.

The size of newly assembled chloroplast genome of *L. praecox* is 152,818 bp, including a large single-copy (LSC) region of 93,756 bp and a small single-copy (SSC) region of 18,910 bp separated by a pair identical inverted repeat (IR) region of 20,076 bp each. A total of 129 genes were successfully annotated containing 85 protein-coding genes, 36 tRNA genes and 8 rRNA genes. GC content of the whole genome, IRs, LSC and SSC regions are 39.1%, 44.4%, 37.9% and 33.9%, respectively. GC content of IRs region is the highest. 20 genes contain one intron, while 2 genes have two introns.

Compared to the chloroplast genome of *L. praecox* collected from Japan (Genbank accession no. MG581449), the China collection was 90 bp longer than Korea collection, 55 bp, 9 bp and 17 bp longer in LSC, SSC and IR regions, respectively. Totally, 220 variations were detected between the two plastomes, including 152 (69.1%) nucleotide substitutions and 68 indels. Among these mutations, 128 (58.2%) located in inter genic regions (IGRs), 88 (40%) in protein-coding regions (22 in intron) and four in tRNA genes’ intron. In summary, almost all of the mutations (66/68) in coding regions were substitution mutations, while 76/128 (59%) substitutions and 52/128 (41%) indels in IGS, 13/26 (50%) substitutions and indels respectively in intron. The comparison results indicated higher intra-species diversity such as *Panax ginseng* and *Lonicera japonica* (Kim et al. 2015; Kang et al. 2018).

The complete chloroplast genome sequence of *L. praecox* and other species from Lauraceae were used to construct phylogenetic tree (Figure 1). The sequences were initially aligned using MAFFT (Katoh and Standley 2013) and then visualized and manually adjusted using BioEdit (Hall 1999). Take the plastome of *Calycanthus floridus* var. *glaucus* (GenBank: NC_004993) as an out-group, a maximum likelihood analysis was performed with RAxML version 8 program (Alexandros 2014) using 1000 bootstrap. IQ-tree was also used to construct ML tree with fast mode (Nguyen et al. 2015). As expected, the result shows the chloroplast sequences of *L. praecox* from China and Japan were clustered together though the two sequences were different, and the position was consist with previous published topology (Jo et al. 2019). The results will be valuable for the genetic diversity study for this species.

**Figure 1.**
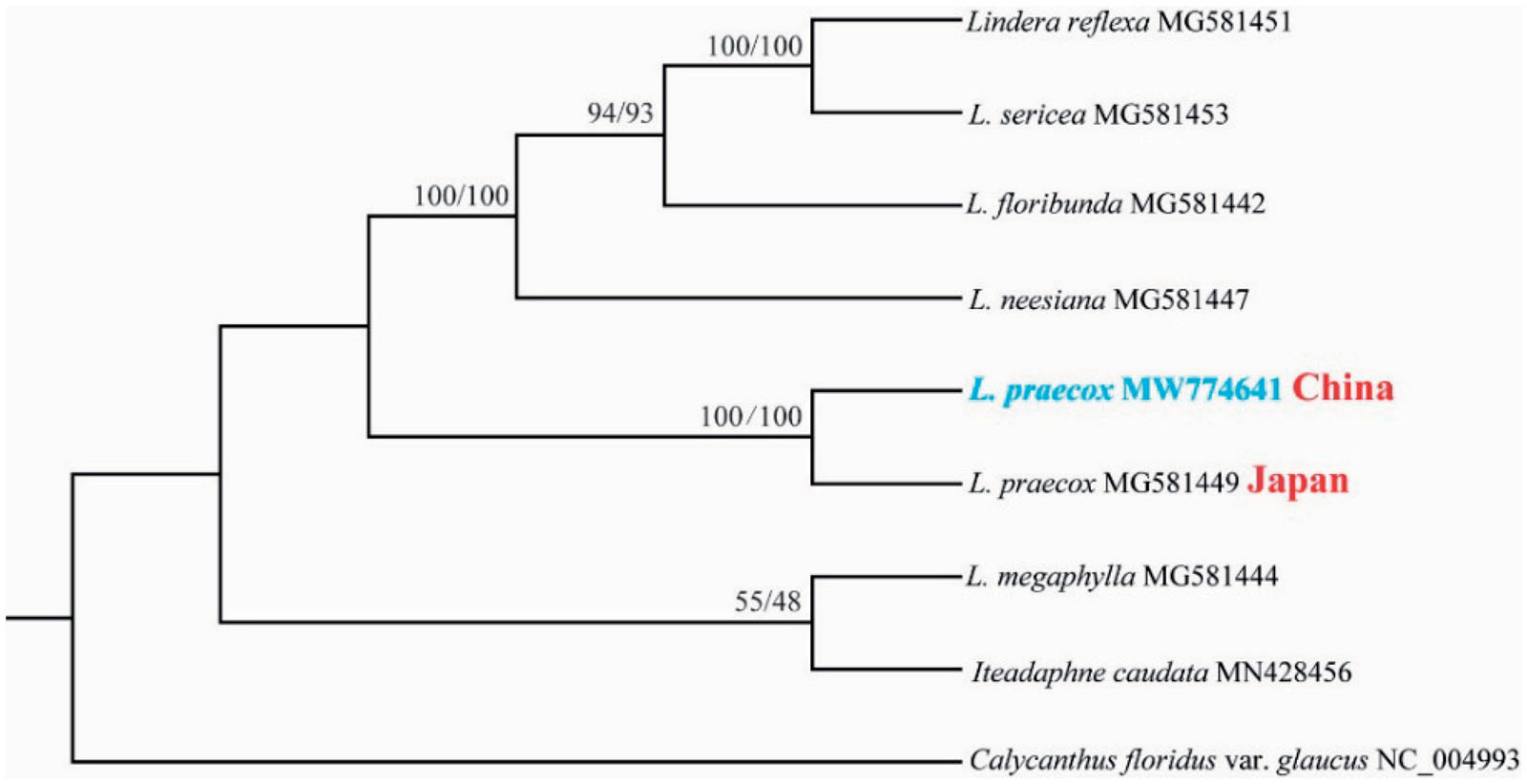
Maximum likelihood phylogenetic tree for *Lindera praecox* based on complete chloroplast genomes. The number on each node indicates bootstrap support value generated by RaxML/IQtree.

## Data Availability

The data that support the findings of this study are available in GenBank of NCBI at https://www.ncbi.nlm.nih.gov, accession number MW774641. The assembled individual was linked with no. SAMN18324953 and Project ID: PRJNA715043.
